# Uniportal Subcostal Video-Assisted Thoracoscopic Surgery: A Feasible Approach for a Challenging Middle Lobectomy in an Obese Patient

**DOI:** 10.1155/2019/5906295

**Published:** 2019-02-07

**Authors:** Samer Al Sawalhi, Deping Zhao, Haomin Cai, Yuxing Jin

**Affiliations:** Department of Thoracic Surgery, Shanghai Pulmonary Hospital, Tongji University, Shanghai, China

## Abstract

Subcostal access is a novel approach for anatomical lung resection. To perform surgery via this access, specially designed long instruments are required. Subcostal access provides excellent visualization of the mediastinum and anterior lung hilum. We exhibit here a subcostal middle lobectomy with systematic en-block mediastinal lymphadenectomy in an obese 52-year-old male patient with body mass index (BMI=37.7) performed via this single incision. The operation was completed efficiently within 30 minutes with negligible postoperative pain.

## 1. Introduction

Thoracic surgical access has moved towards minimally invasive methods. The presumed advantages of this are lesser tissue trauma, swift recovery, minor wound infection, lower pain scores, and reduced hospital stay. However, there are also reports of prolonged port-site pain with single or multiple video-assisted thoracoscopic surgery (VATS) [1]. Moreover, single-port thoracoscopic surgery could also cause a similar problem. Liu et al. [2] reported first subxiphoid uniportal VATS left upper lobectomy on 2014 and hence subcostal is proposed as an alternative access to dwindling intercostal pain and neuralgia by avoidance damage of the intercostal nerve. This technique is more challenging due to the instruments-fighting problem. That is why it required long, curved, and articulated instruments to deal with upper, lower lung hilum and mediastinum. The mean BMI reported in the literature for the subxiphoid technique is around (22.0±0.83) [3]. This case is unique due to the high BMI and we propose that it is a safe technique in expert hands. We discuss the pros and cons of this approach for the first time in an obese patient.

## 2. Case Report

**Figure 1:**
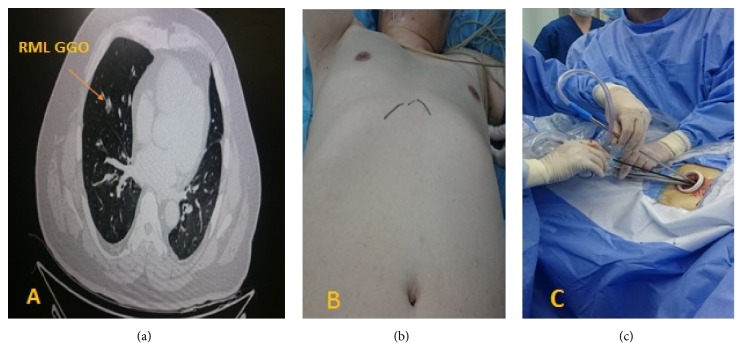
(a) Chest C-T scan delineates the right lung middle lobe with a solid GGO measuring 1.5 cm in size. (b) Dorsal decubitus patient position with 40° angle during subcostal approach for right middle lobe lung lobectomy. (c) Subcostal oblique incision (5 cm) parallel to right costal arc and covidien wound protector with arrangement of instruments through the port.

We report a 52-year-old gentleman, asymptomatic, and nonsmoker, without comorbidities but with class II obesity BMI of 37.7. He was referred from the lung cancer screening program as the high-resolution chest computed tomography (CT) scan delineated a 1.5 cm mixed ground glass opacity (GGO) with a larger solid component in the right middle lobe (RML) (Figure 1(a)). This nodule increased in size at the follow-up CT scan; therefore we decided to resect the middle lobe anatomically using subcostal access.

### 2.1. Procedure

**Figure 2:**
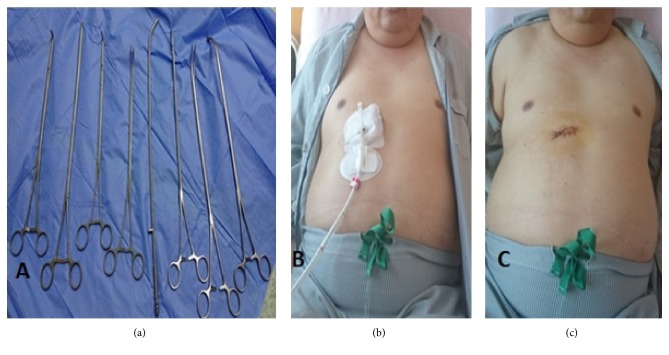
(a) Long curved instruments were recommended to use in subcostal approach. (b) First postoperative day in an obese class II patient with chest tube inserting through subcostal wound. (c) 5 cm subcostal incision after removing chest tube.

The patient was given general anesthesia with double lumen endotracheal intubation and placed in a lateral decubitus position (dorsal decubitus 40 degrees) (Figure 1(b)). A 5 cm oblique incision parallel to the costal arch was made. Then the subcutaneous tissue and rectus abdominis were dissected along the subcostal margin; xiphoid process and pericardiophrenic fatty tissue were detached. A subcostal tunnel was bluntly dissected using finger until the mediastinal pleura was opened at the cardiophrenic angle. Lastly, covidien wound protector (WPLGR914) was placed (Figure 1(c)). A thoracoscopic lens with a 30° angle (Olympus, Melville, NY) was used and interlobar fissure was examined. Lobectomy was performed as in the video [subcostal right middle lobe lobectomy video] (available here). Systematic mediastinal lymph nodes dissection includes stations 2,4 right paratracheal, 9,10,11,12 and subcarinal lymph nodes. Long and sturdier VATS instruments were used. Also, curved tip stapler technology aids to allow passage around the vascular structures (Figure 2(a)). We did not put any extra ports and the operative time was 30 minutes.

After completing the procedure, a 28 F chest tube (Figure 2(b)) was inserted through the same subcostal wound. The patient was given routine venous thromboembolism prophylaxis (6000 i.u UFH) 12 hours after operation. No postoperative arrhythmia or air leak was detected. The patient was discharged on the third postoperative day with an uneventful recovery (Figure 2(c)). Postoperative pain score (Wong-Baker FACES) on 1, 2, 3,7,15 days was evaluated. It was (4-2-0-0-0) consecutively. During the follow-up, no incisional hernia was detected. The pathology results delineated a (1x 0.8) cm invasive infiltrating adenocarcinoma [80% ductal/20% papillary] without lymphovascular, neural, or pleural invasion (T1aN0M0), stage I A1 according to the non-small-cell lung carcinoma (NCCN) guidelines version 4.2018-8th edition]. Five lymph nodes were harvested, and all were negative for metastasis (0/5).

## 3. Discussion

Nowadays, we noticed that surgery has moved towards gentleness for more than a century, with minimally invasive surgery instructing us to treat tissue gently. The subcostal uniportal VATS lobectomy is an innovative modification of VATS. Several studies and meta-analyses have suggested a long-term oncological benefit of VATS lobectomy in the radical treatment of patients with NSCLC in the early stages of the disease [4]. The indications for using the uniportal subcostal approach included the presence of early stage T1 or T2 tumor (<5 cm), N0 status, and thymectomy. Furthermore, it is a good option to deal with bilateral metastasectomies, bilateral lung lesions, and bilateral lung bulla resection in bilateral pneumothorax.

Those patients who are vulnerable for subcostal approach should be selected carefully, especially those having a tumor, or GGO (solid, pure, or mixed) less than 2 cm in maximum diameter and they do not have suspicious of mediastinal lymph nodes metastasis. Moon Y et al. [5] delineated in his study that mediastinal lymph nodes evaluation is not mandatory for clinical N0 NSCLC presenting as a 3 cm or smaller GGO-predominant.

Exclusion criteria for using the uniportal subcostal approach were chest wall tumor involvement, central masses, previous thoracic surgery, adhesion, body mass index BMI>30 kg/m^2^ [3, 6, 7], cardiomegaly, cardiac comorbidities due to compression on the heart specially on the left side, and enlarged lymph nodes with confirmed N1 or N2 disease [7].

We concluded from our previous research which included 77 patients with lung cancer who underwent mediastinal lymph nodes sampling or dissection that there were no significant differences in lymph node dissection between subxiphoid and intercostal groups [8]. We chose subcostal access in this patient due to the following reasons:

(1) In obese patients uniportal VATS lung resection may be arduous, as the thickness of the subcutaneous fat in the chest wall makes the manipulation of instruments through the port much more difficult.

(2) Narrow intercostal spaces in obese people will exacerbate more entrapment of intercostal neurovascular bundle which leads more postoperative neuropathic pain.

(3) Subcostal incision is a “true” thoracic wound; i.e., the surgical wound is below the sternocostal triangle and above the diaphragm; therefore, herniation of viscera through the incision is rare.

The advantages of this approach for anatomical lobectomies or segmentectomies are shortening the operation time and blood loss especially in patients who got bilateral lung lesions at the same time. Moreover, no need to change the patient position during the surgical operation.

As the subcostal incision is away from the breast, it is cosmetically more acceptable for women. The presence of excessive fatty tissue, like in our patient, may pose a burden to this procedure (Figure 2(b)). Furthermore, chronic postoperative intercostal pain and neuralgia were significantly lower in the subcostal approach [2]. Upon our experience and our previous research [8] within this approach, we found that oblique incision is much better regarding field exposure and instruments manipulation. Therefore, we suggest an oblique incision for unilateral operations and transverse incisions for bilateral operations in the subxiphoid uniportal VATS.

It was reported that patients with obesity are not good candidate for subcostal approach [7]; therefore the challenging point also in obese patients is high location of the diagram, because of the abdominal pressure effect; therefore we compromise that, by resection both xiphoid process, mediastinal and pericardial fatty tissue, these steps allow enlarging the space for instruments manipulation.

For a routine lung cancer resection, a minimum of three N2 mediastinal lymph node stations should be sampled or complete dissection, based on the NCCN Guidelines version 4.2018 non-small-cell lung cancer 8th edition. Liu et al. [2] reported that systemic lymph nodes dissection through the xiphoid incision was difficult. Systematic lymph nodes dissection including the lymph nodes of the left group 4L,5,6,7,8,9,10,11 and the right group 2,3a,4R,7,8,9,10,11 should be carried on by an expert surgeon with a subcostal approach to avoid complications through traction of the lung and the coordinated reversal of the operation table [8].

Tips, tricks, and recommendations for mediastinal lymph nodes dissection are as follows:

(1) Using subcostal oblique incision facilitates access to subcarinal lymph nodes [8].

(2) Using thoracoscopic lens with a 30° angle or articulated lens gives good visualization for subcarinal space.

(3) Releasing inferior pulmonary ligament enables easy access to subcarinal space.

(4) Energy devices like Harmonic Scalpel (Ethicon, Inc.) can be used for dissection and maintaining hemostasis at the same time [7].

(5) Proper traction of the lung anteriorly and medially facilitates exposure subcarinal space.

(6) Superior vena cava was retracted and the azygos vein was pulled up using a suction device to harvest paratracheal lymph nodes.

The instrument-fighting problem during the subcostal single-port surgery would be even more challenging than the transthoracic approach since the mediastinum occupies the lower part of the working tunnel, which reduces the working space. We used a small tidal volume to let the mediastinum drop into the dependent nonoperative side. The surgeon practicing subcostal uniportal VATS needs previous experience in uniportal VATS lobectomy and skilled assistant [6].

The drawback of subcostal approach is difficult to control bleeding, once it happens because you are working from a small window; therefore our advice in such situation is to compress the bleeding site by pillar gauze and convert to thoracotomy immediately. Furthermore, this approach required specific designed long instruments like curved ring forceps, Harmonic Scalpel, curved long suction, long DeBakey forceps, and long electrocautery hook, in addition to long, curved articulated stapler technology to enable passage around the vessels and bronchus smoothly.

Subcostal approach is easier on the right side than on the left side, because of the presence of beating heart and incidence of arrhythmia while compressing the heart by instruments manipulation. Expert thoracic surgeons with a skilled assistant allow performing more sophisticated kinds of challenging operations using a subcostal approach like [9]; lobectomy in an obese patient, calcified lymph nodes, and fissure less lobe.

The cornerstone of choosing the surgical approach is the achievement of complete radical resection (R0) and efficient mediastinal lymph nodes dissection.

It is worth considering the subcostal approach in patients who are a candidate for it, taking into consideration the safety and the oncological principles.

Finally, in an era of robotic surgery, using uniportal subcostal access is promising in such cases, once articulated instruments will add more help in the procedure and lymph node dissection.

## Supplementary Materials

Supplementary Materials Uniportal subcostal video-assisted thoracoscopic surgery, a feasible approach for a challenging middle lobectomy in an obese patient. Subcostal approach is proposed as an alternative access due to avoidance of damage intercostal nerves. The cornerstone of choosing the surgical approach is the achievement of complete radical resection (R0). The patient was given general anesthesia with double lumen endotracheal intubation and placed in a lateral decubitus position (dorsal decubitus 40°). A 4 cm oblique incision parallel to the costal arch was made; then the subcutaneous tissue and rectus abdominis were dissected along the subcostal margin. A subcostal tunnel was bluntly dissected using finger until the mediastinal pleura was opened at the cardiophrenic angle. We used electrocautery to dissect the mediastinal pleura to get access to the chest cavity; covidien wound protector (WPLGR914) was placed. A thoracoscopic lens with a 30° angle was used and interlobar fissure was examined. Interlobar vein was cut after applying hemlock clip using (Harmonic Scalpel (Ethicon, Inc.)) knife. Long and sturdier VATS instruments were used. Also curved tip stapler technology aids to allow passage around the vascular structures. We started dissection of middle lobe vein and stapling using endovascular vascular cartridge [Medtronic, Minneapolis, MN, USA]. Middle lobe bronchus was dissected with endoscopic green stapler. Peribronchial lymph nodes were harvested. Two middle lobe arteries were stapled by endovascular vascular cartridge. Finally, the fissure was stapled and the lobe was harvested by endobag TKBAG HSD-130 (Guangzhou T.K Medical Instrument Co., Ltd.). Systematic mediastinal lymph nodes dissection including stations 2,4 right paratracheal and subcarinal lymph nodes after releasing the inferior pulmonary ligament and traction of the lung anteriorly and medially facilitates exposure subcarinal space.
